# Antemortem diagnosis and prevention of human rabies

**DOI:** 10.4103/0972-2327.40219

**Published:** 2008

**Authors:** Shampur Narayana Madhusudana, Suja Moorlyath Sukumaran

**Affiliations:** Department of Neurovirology, Human Brain Tissue Repository, National Institute of Mental Health and Neurosciences (NIMHANS), Bangalore - 560 029, India; 1 Department of Neuropathology, National Institute of Mental Health and Neurosciences (NIMHANS), Bangalore - 560 029, India

**Keywords:** Diagnosis, polymerase chain reaction, rabies

## Abstract

Human rabies still continues to be a significant health problem in India and other developing countries where dogs are the major vectors of transmission. Rabies in humans can present in two clinical forms, i.e., furious and paralytic. While diagnosis of furious rabies can be made based on the typical symptoms and signs, paralytic rabies poses a diagnostic dilemma to the neurologists who may encounter these cases in their practice. Although there are certain clinical features that distinguish this disease from other forms of Guillain-Barre syndromes, confirmation of diagnosis may require laboratory assistance. Conventional techniques such as antigen detection, antibody assays and virus isolation have limited success. The recently introduced molecular techniques show more promise in confirming the cases of paralytic rabies. There has not been much success in the treatment of confirmed rabies cases and recovery from rabies is extremely rare. Therefore, preventive measures of this dreaded disease after an exposure become extremely important. The present article reviews the current status of human rabies with regard to antemortem diagnosis, disease management and post-exposure prophylaxis.

## Introduction

Rabies - the oldest and most feared human disease known to man - causes an acute, progressive incurable encephalomyelitis caused by highly neurotropic ssRNA virus, taxonomically classified in the genus Lyssa virus and family Rhabdoviridae. Rabies in humans can present in two clinical forms, i.e., furious and paralytic. While the diagnosis of furious rabies can be made on distinctive symptoms and signs, paralytic rabies poses a diagnostic dilemma to the neurologists. Although there are certain clinical features that distinguish this disease from various forms of Guillain-Barre syndromes, confirmation of diagnosis may require extensive laboratory assistance. Neurologists in developing countries are likely to encounter cases of rabies encephalitis, particularly atypical and paralytic forms and a few with psychiatric manifestations for many years to come. An array of laboratory tests has been carried out in laboratories for the diagnosis of rabies, each having its own merits and de-merits. Conventional techniques such as antigen detection, antibody assays and virus isolation are time consuming and have limited success. In the present era, the antemortem diagnosis of rabies is gaining tremendous attention. For instance, the survival of patients presenting with hydrophobia are being reported from various geographical locations around the globe and in developing countries such as India.[1,2] There is an increasing need for the institution of control measures since human rabies still continues to be a significant health problem, where stray dogs are the major vectors of transmission. Recently, molecular biological techniques are introduced for confirming the diagnosis of paralytic rabies and these are quite promising. Therefore, antemortem testing for rabies is gaining significance since a delay in the diagnosis can culminate in the contamination of instruments in intensive care units and unnecessary requirement for post-exposure prophylaxis for bystanders and nursing staffs. In the recent past, several advances have been made in the field of rabies, particularly in understanding the molecular biology of the virus, molecular epidemiology, pathogenesis and prevention of the disease. More efforts have to be focused on antemortem diagnosis and management of this dreaded rabies infection. The purpose of this review article is to highlight some of these aspects that will be of interest to a neurologist in his day-to-day practice with emphasis on diagnostic dilemmas faced with atypical and paralytic rabies cases, newer diagnostic modalities available, recent efforts in treatment of the disease and state of the art preventive measures.

### The virus

**Figure 1:**
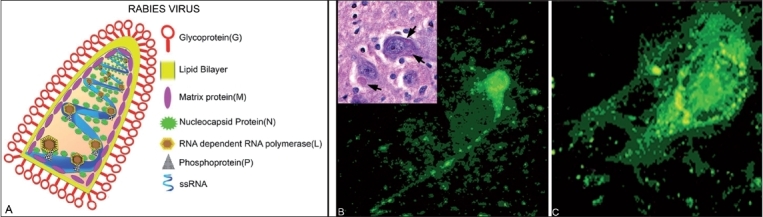
(A) Schematic representation of internal structure of rabies virus and the position of various viral proteins. (B) Direct immunofluorescence staining on a fresh human brain smear with polyclonal antibody to nucleocapsid tagged to FITC bright greenish yellow fluorescent rabies antigen particles in side the neuron and along the axons. ×600. Inset: Eosinophilic intracytoplasmic Negri bodies in neuronal soma ×360. (C) BHK 21 cells infected with CVS strain of rabies in RFFIT. ×600

Rabies virus is a negative sense single stranded RNA virus belonging to the genus Lyssavirus and family Rhabdoviridae. It is a bullet-shaped enveloped virus measuring 180 nm × 75 nm. The envelope is surrounded by numerous spikes made up of glycoprotein (G), which are necessary for viral attachment to receptors and is also a major protein for the induction of neutralizing antibodies. The other important proteins are the ribonucleoprotein (N), which is intimately associated with the helical RNA, the phosphoprotein (P) and a matrix protein (M) [Figure 1A]. The rabies genome has approximately 12,000 nucleotides. Seven genotypes of the virus have been identified from different parts of the world. In India and other Asian countries, only genotype 1 is prevalent.

### Pathogenesis and pathology

Human infection by the rabies virus almost always occurs through the bite of an infected animal, dogs being the major vector of transmission. Direct transmission through the nervous system from the site of bite can occur in which case the incubation period tends to be short, sometimes as short as a week, if there are sufficient nerve endings exposed at the wound site. However, in majority of the cases, the virus undergoes a period of replication in the exposed muscles before entering the motor or sensory nerves supplying the muscle.[3] Nicotinic acetylcholine receptors have been identified as the major receptors for rabies virus.[4] After initial replication, the virus ascends in the axoplasm of the nerves to the spinal cord and then to the brain where rapid multiplication takes place involving almost all the areas of the brain. The initial involvement of hippocampus, hypothalamus and areas of the limbic system can explain some of the dramatic clinical features. In spite of the extensive replication of the virus in the brain, pathological findings are minimal. The only specific and diagnostic pathological feature is the presence of Negri bodies. Recent evidence has shown that the defective functioning of neurotransmitters in the brain may play a role in the pathogenesis.[5] Some studies have suggested that neuronal apoptosis rather than necrosis plays a role in the pathogenesis of rabies leading to fatality.[6]

## Clinical Features

The development of rabies encephalitis in an exposed individual depends on several factors viz. the severity of bite, site of bite, virus content in the saliva and probably some non-specific host immune factors. Transdermal bites on the head, neck and hands carry the highest risk and are usually associated with shorter incubation period. However, bites on the lower extremities are more common, and as revealed in the recent Indian survey, 80% of all human rabies cases had bites on the legs. It is also known that not every rabid bite results in clinical rabies. Rabies mortality varies from 35 to 57% in unvaccinated bite victims.[7] After a variable incubation period, the patient either develops classical and more frequently seen furious rabies or less common paralytic (dumb) rabies.

### Incubation period

The incubation period for rabies is the most variable of all acute CNS infections. The average incubation period is around 1-3 months but may range from less than 7 days to 6 years. It is not still clear why there is such a variable and prolonged incubation period. The incubation period may depend on several factors including density of rabies virus receptors in the affected tissue, the degree of innervation in tissues, the quantity of virus inoculated and the properties of the rabies virus stain.[8] It is assumed that cases with unusually prolonged incubation period may be due to a trivial exposure considered insignificant and therefore not disclosed. It is also possible that there may be no history of bite in some cases of human rabies. In our experience at NIMHANS from 1993 to 2004, 2 cases out of 37 autopsy confirmed rabies cases gave no history of animal bite. In Thailand, nearly 6% of 707 cases of human rabies in the past two decades had no history of bite.[9]

### Prodromal symptoms

Except for the local symptoms at the site of bite or bitten extremity, these are usually vague and nonspecific. There may be fever, malaise, generalized mayalgia and a sense of “not well-being.” In nearly 50% cases of paralytic rabies and 30% cases of furious rabies, local manifestation in the form of itching, pain or paresthesia at the site of bite may be the earliest symptom, which is often neglected by the patient and ignored by the physician. This symptom may later involve the entire bitten extremity. The sensory function is usually intact, and at this stage, there is no demonstrable weakness of the bitten extremity.

### Acute neurological phase

Within hours or a few days after the prodrome, rabies patients enter an acute neurological phase. Two-thirds of patients suffer from an encephalitic or furious form and the remaining present with paralysis or a condition resembling Guillain-Barre syndrome (GBS). Patients with encephalitic form of illness usually die within 3-4 days; however, there may be a prolonged course of illness, sometimes up to 15 days in cases of paralytic rabies, especially if these patients are treated in intensive care units. A case of paralytic rabies at the neurological services of national institute of mental health and neurosciences (NIMHANS), Bangalore, India survived up to 45 days under these conditions.

### Furious rabies

The earliest manifestation of furious rabies is nervousness and hyperactivity resembling an acute anxiety reaction, often associated with moderate to high-grade fever. Initially, mentation is preserved, but the attention span is shortened and within hours three typical features of furious rabies develop: phobic spasms, fluctuating consciousness and signs of autonomic dysfunction.

Typical phobic spasms include hydrophobia and aerophobia. These are seen in almost all patients with furious rabies, although they may not be present in later stages of the disease when drowsiness and coma supervene. The term “hydrophobia” is derived form the Greek word meaning “fear of water.” This term is almost synonymous with rabies in humans. Aerophobia and hydrophobia can be demonstrated by blowing or fanning of air on the face or chest wall and by asking the patient to swallow water or mere offering a glass of water. Intense startling reaction results from spasms of the accessory respiratory muscles of the neck, pharyngeal muscles and diaphragm followed by extension of the neck and a feeling of dyspnoea. During these episodes, they are extremely aroused and exhibit fearful facial expressions. The pathophysiological mechanism of hydrophobia, which is only observed in humans and not in rabid animals, is still not clear. The mental status alternates between periods of agitation and apparent normal mental status. As the disease progresses, confusion becomes severe and patients can become wild and aggressive. The period of agitation is gradually followed by impaired consciousness and coma.

The signs of autonomic dysfunction include hyper salivation, pupillary abnormalities such as constricted or dilated pupils, anisocoria, generalized piloerection, excessive sweating, priapism and rarely spontaneous ejaculations. Occasionally patients may present with typical features of mania, increased sexual arousal, attempts to rape, all of which can make the patients to land in psychiatric clinics.[10]

### Paralytic (dumb) rabies

This form of rabies is seen in approximately 20% of patients, often with diagnostic problems. The major cardinal signs of furious rabies may never appear or appear very late in the disease and can be very mild. Weakness usually starts in the bitten extremity and then progressively involves all limbs and pharyngeal and laryngeal muscles. Facial weakness is a common feature. Some of the features that may differentiate paralytic rabies from Guillain Barre Syndrome are:

1. Fever is almost always present in paralytic rabies but usually absent in cases of GBS once weakness develops, unless followed by complications such as aspiration pneumonia.
2. Sensory function is intact in paralytic rabies, except for the initial paresthesia at the site of bite.
3. Quadriparesis with the predominant involvement of proximal muscles and urinary incontinence are always found in the early course of paralytic rabies. In cases of GBS, urinary incontinence is rare. In GBS, especially in the early phase, both proximal and distal muscles are affected.
4. Percussion myoedema may be present in both early and later stages of paralytic rabies. This is best elicited by the percussion of the chest, deltoid or thigh region and consists of mounding of a part of the muscle at the percussion site, which then flattens and disappears over a few seconds. It is not observed in patients with GBS, encephalitic form of rabies or in patients with neurological complications following Semple type of sheep brain rabies vaccines.
5. Generalized or focal fasciculations are frequently present in paralytic rabies patients.

Hydrophobia is unusual and can be terminal manifestation in the paralytic form of the disease. Survival is usually longer than in the furious form. Eventually, bulbar and respiratory muscles are paralyzed resulting in death due to cardio-respiratory arrest. Although the hallmark of paralytic rabies is ascending paralysis, sometimes the features of furious rabies such as anxiety, aerophobia, inspiratory spasms and altered sensorium may be observed - particularly in the terminal stages - thereby resulting in the overlap of symptoms between the two forms. These features, if present, provide a clue to the diagnosis of rabies rather than GBS.

The pathogenetic basis for the two different clinical forms of rabies is not known. There is no difference in the virus strains that cause furious and paralytic forms of the disease and has no correlation with the incubation period or the site of bite. However, paralytic rabies is more frequently observed in patients who take partial course of either Semple or cell culture rabies vaccines. It is possible that paralytic rabies results from an immunopathologic attack on virus infected cells in the brain and spinal cord.[11]

## Clinical Investigation - MRI Imaging

Computed tomographic (CT) studies of the brain are usually normal in both forms of rabies, although hypodense lesions in cortex and basal ganglia have been described.[12] On Magnetic Resonance Imaging (MRI) of the brain, both normal features and increased signal intensities in grey matter areas have been described.[13] Antemortem MRI has shown predilection for the involvement of brain stem, hippocampus and hypothalamus in both the clinical forms. The MRI in five cases (2 furious and 3 paralytic) demonstrated ill-defined hyperintensities on T2-weighted images in the brain stem, hippocampi, hypothalamus, subcortical and deep white matter and cortical grey matter as early as day 3 of onset even when the consciousness was preserved.[14] Contrast-enhanced lesions were observed in the brain stem, hypothalami and spinal nerve roots only when patients became comatose. The spinal cord anterior horn was found involved in both clinical forms of rabies on gradient echo T2-weighted images.

## Laboratory Investigations

The electroencephalogram (EEG) may be normal or show nonspecific abnormalities in human rabies. Slow wave activities as well as periodic epileptic form activities have been observed.[15] Hematological and biochemical studies are usually normal. The cerebrospinal fluid (CSF) analysis may be normal or may show pleocytosis and mild elevation of protein concentration.

Serum, CSF, saliva and tissue such as skin, from highly innervated anatomical areas are the appropriate biological specimens for antemortem diagnosis and all these samples should be considered as potentially infectious see [see Table 1]. The appearance of high titer of antibodies to specific viral antigens by serum neutralization tests is diagnostic in a patient with encephalitis, with no history of preimmunization. Rabies specific antibodies usually appear after 7 days of the illness in an unvaccinated patient and may be a good diagnostic marker in cases with paralytic rabies who usually survive longer.[16] In paralytic rabies, both serum and CSF antibodies may be present and the demonstration of significant levels of antibodies in CSF can differentiate paralytic rabies from encephalomyelitis, resulting from administration of Semple's antirabies vaccine.[17] However, the presence of antibodies in serum and CSF in an already vaccinated patient should be interpreted with caution, and a confirmatory diagnosis can be made only after the demonstration of significant rise in antibody titer between the first and the second sample collected after 7-10 days.

**Table 1:** Availability of Antemortem diagnosis of human rabies

Tests	Specimens	Specificity %	Sensitivity %	Remarks
DFA on corneal smear (antigen)	Corneal smear	90	30	Not very sensitive
DFA on skin biopsy (antigen)	Nuchal skin	100	50-70	More sensitive than corneal test
RT-PCR on saliva for viral nucleic acid	Saliva	100	50-70	Moderate sensitivity
Real time PCR on saliva for viral nucleic acid	Saliva	100	70-80	Higher sensitivity
Virus isolation from saliva by RTCIT	Saliva	100	70-80	Time consuming
Antibody detection* in Serum/CSF by RFFIT	Serum and CSF	100	70	Time consuming

* Should be interpreted based on history of prior vaccination; DFA - Direct Fluorescent Antibody Test; RT-PCR - Reverse transcriptase-polymerase chain reaction; RTCIT - Rabies Tissue Culture Infection Test; RFFIT - Rapid Fluorescent Focus Inhibition Test

Viral isolates can be obtained by animal inoculation or cell culture passage of saliva or oral swabs, although of limited utility in clinical setting. These samples can be probed for viral nucleic acid. Viral antigen can be detected by Direct Fluorescent Antibody (DFA) test on brain biopsy material collected usually during *post mortem* to establish a definitive diagnosis [Figure 1B], corneal touch impressions or a full-thickness nuchal skin biopsy from the hairy nape of the neck.

### Corneal smear test by fluorescent antibody test (FAT)

Rabies virus antigen can be detected by a simple FAT test on corneal epithelium during the terminal stages of the disease. *Intra vitam* diagnosis of rabies by Fluorescent antibody test (FAT) in corneal impression was first described by Schneider in animals and by Cifuentes *et al.* in humans.[18,19] The methodology of this test involves the gentle rubbing of flat surface of a clean microscope glass slide on each cornea. Samples are taken with at most care because of the risk of permanent damage to the cornea. The smear, as usual, is fixed in acetone and subjected to routine FAT. In general, the antigen appears characteristically as round to oval intracytoplasmic inclusions in corneal epithelial cells. The sensitivity of the test is comparatively low and depends on the stage of the disease. Mathuranayagam *et al.* showed that the reliability of corneal impressions for rabies diagnosis is limited, especially when sampling is performed under field conditions and a negative result could not rule out the diagnosis of rabies.[20]

### Nuchal skin biopsy

The examination of skin biopsy material was shown to be a valuable technique for *intra vitam* diagnosis in animal and humans.[21,22]

Blenden *et al.* found that 25-50% of patients showed immunofluorescence positive results for rabies viral antigen, and a proportion of positive results during the early phase of clinical illness increased as the disease progressed.[23] The test is simple, and for sample selection, a section of skin that has a diameter of 5-6 mm should be taken from the posterior region of the neck along the hairline. The biopsy section should contain a minimum of 10 hair follicles and must be of sufficient depth till the subcutaneous plane in order to include the cutaneous nerves at the base of the hair follicle. Preservatives or additional fluids should not be added. The tissue as usual is fixed in acetone and subjected to routine FAT on cryosections. The antigen is usually present in the nerve fibres surrounding the base of hair follicles. At least twenty serial sections need to be examined before giving a negative result. The result is not related to the antibody status of the patient. The test becomes more sensitive as the disease progresses. The examination of skin biopsies may also be used for *post mortem* diagnosis in countries, where the opening of skull of the dead person is not accepted on cultural and religious grounds.

### Detection of RNA in saliva and CSF by PCR

**Figure 2:**
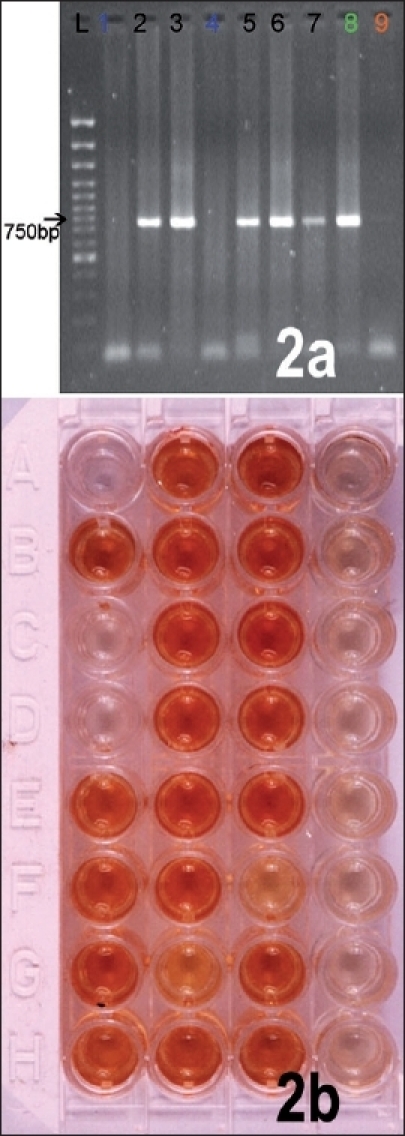
(A) RT-PCR for N gene on saliva samples taken from rabies patients. (B) Rapid rabies Enzyme Immunodiagnosis (RREID). The dark brown colored wells represent brain samples positive for rabies antigen

The demonstration of viral nucleic acid in saliva, CSF, corneal smear, skin biopsy by Polymerase Chain Reaction (PCR) appears to be quite promising for the antemortem diagnosis of human rabies [Figure 2A]. Both conventional Reverse transcriptase Polymerase Chain Reaction (RT-PCR) and Real time PCR (TaqMan) have been described for the detection of rabies. Smith *et al.* have reported the use of hemi-nested RT-PCR for rapid *ante mortem* diagnosis within 36 h of sample submission in case of suspected rabies, where Fluorescent antibody test (FAT), Mouse inoculation test (MIT) and Rabies Tissue Culture Infection Test (RTCIT), have yielded a negative results. Smith *et al.* standardized hemi-nested RT-PCR, followed by automated sequencing and confirmed the presence of classical rabies virus (genotype1) in both saliva and skin specimens with in 36 h.[24] Nagaraj *et al.* evaluated the utility of conventional RT-PCR and SYBR Green Real time PCR for the *ante mortem* diagnosis of rabies using saliva samples. Real-time PCR assay was found to be more sensitive than conventional RT-PCR assay, highlighting the utility of molecular diagnostic tests in establishing *ante mortem* diagnosis of rabies using saliva samples within a few hours.[25] As the secretion of rabies virus into the saliva is intermittent, its isolation from saliva may not always yield successful results. On rare occasions, virus has been isolated from the CSF of patients. Saengseesom *et al.* conducted a study to find presence of rabies virus in saliva and cerebrospinal fluid (CSF) of suspected live rabid dogs at the time of quarantine by using a SYBR Green real-time RT-PCR based assay for the detection of rabies virus RNA. Positive CSF samples were found in 4 out of 15 dogs (27%), whose saliva samples were tested. The time interval from sample collection to result was less than 5 h. Their study suggested that because the virus may be absent or present at very low levels in clinical fluids, samples taken for ante-mortem diagnosis cannot definitively rule out rabies in the animal.[26] In 2001, Wacharapluesadee and Hemachudha described a technique that can be completed in 4 h based on the amplification of nucleic-acid sequences to detect rabies-specific RNA in the saliva and cerebrospinal fluid (CSF) of four living human subjects with rabies.[27] Rabies viral RNA could be detected in either saliva or CSF or both, in all patients as early as day 2 after the onset of symptoms. The isolation of virus from saliva and CSF is influenced by the antibody status of the individual. The success rate is more in early stages when neutralizing antibodies are not present or of low titer.

### Virus isolation

Virus isolation from animals (or humans) is one of the most reliable methods for the diagnosis of rabies. Virus isolation from infected tissue can be carried out either in mice (mouse inoculation test - MIT) or in cell culture (rabies tissue culture isolation test-RTCIT). Rudd *et al.* proposed the tissue culture technique (BHK 21 cells) as a routine method for the isolation of street strain of rabies virus.[28] The specimens collected *intra vitam* includes saliva samples, throat swabs, swabs of the nasal mucosa, corneal smears and cerebrospinal fluid.[29]

Mouse inoculation test (MIT)

The Swiss albino mouse is preferred for rabies diagnosis, but other strains can also be used since all are equally susceptible to intracerebral inoculation with street rabies virus. The Swiss albino mice are the most susceptible and common animals used for virus isolation. While weanling mice {21 to 30 days old, (weighing 9-15g)} are preferred, suckling mice less than 4 days are more susceptible. The homogenized brain tissue sample in buffered diluent containing protein stabilizer and antibiotics are intracerebrally inoculated (0.03 ml) into a minimum of six weanling mice for each specimen.

The technical simplicity and high sensitivity are favored attributes of MIT. The main drawback of this test is the technical expertise, environment and ethical issues related to the usage of large number of animals.

Rapid tissue culture infection test (RTCIT)

The inoculation of the cell culture is a recognized method for the isolation of street virus for rabies diagnosis at present. The unpredictable and problematic delay with *in-vivo* virus isolation is considerably reduced with the cell culture inoculation. Several cell lines may be used for the isolation such as BHK 21, the CER and mouse neuroblastoma.[30,31] The specimens routinely used for virus isolation include brain and /or salivary glands, suspensions of which are prepared and inoculated into the cultures. After overnight incubation (18 h) at 37°C in 5% CO_2'_, the cells are fixed, stained with an antinucleocapsid antibody conjugated to fluorescein isothiocyanate (FITC) and observed under a fluorescent microscope.[29] In many laboratories, the RTCIT has replaced the MIT as it is relatively easy to perform, cost-effective and can substantially reduce the time required for obtaining results.

### Detection of antibodies from CSF and serum

An indirect evidence for rabies infection is demonstrating a high titer of antibodies in CSF in nonvaccinated individuals. The following are the WHO (World Health Organization)-recommended tests based on this principle: Mouse Neutralization Test (NT) and Rapid Fluorescent Focus Inhibition Test (RFFIT).

Mouse Neutralization Test (MNT)

Webster and Atanasiu developed virus neutralization test in mice (mouse neutralization test, (MNT) in 1935.[32,33] This is a widely used WHO recommended test and has become the standard against which other tests are now evaluated. The challenge virus for the test is titrated by intracerebral inoculation of 21-day-old weanling mice. The dilution of virus that kills 50% of inoculated mice, i.e., LD_50'_, is determined by the Reed-Muench or Spearman-Karber methods. To each serum dilution from a suspected case of rabies, 32 to100 times the LD_50_ virus is added and the mixture is incubated for 90 min at 37°C and then inoculated intracerebrally into weanling mice (5-10 mice for each serum dilution). The mice are observed for 14 days, and the number of deaths occurring in each serum dilution is noted. The dilution of serum at which 50% of the mice are protected (i.e., survived) is calculated by the Reed-Muench or Spearman-Karber methods. The precision of the assay is limited by the number of mice inoculated for each serum dilution and the increment between dilutions to be used. Tediousness and economics limit the numbers of mice, to be inoculated and dilutions to be evaluated. The individual resistance and nonspecific mortality associated with any animal test also adversely affects the precision of the test.[34]

The adaptation of the “Challenge virus standard (CVS) strain” of rabies virus (attenuated virus) to grow in cell culture has led to the development of alternative assays in which the replication of non-neutralized virus in culture is detected by the observation of plaques, fluorescent antibody-stained infected cells or the colour developed with enzyme-labeled anti-rabies antibody

Rapid Fluorescent Focus Inhibition Test (RFFIT)

The RFFIT is one of the most widely used substitutes to the Mouse Neutralization Test (MNT) and was developed by Smith *et al.*[35] It is a rapid test that requires only 20 h for completion. It involves the observation of foci of virus-infected cells by fluorescent antibody staining [Figure 1C]. The sera are serially diluted in 8-well Lab-Tek slides, followed by the addition of 30-100 FFD_50_ (Focus Forming Dose; One FFD_50_ is the dilution of virus at which 50% of the observed fields contain one or more infected cells) of virus and incubating the serum-virus mixture at 37°C. Subsequently, suspensions of BHK-21 cells are added. After a 20-h incubation period at 37°C in a 5% CO_2_ incubator, the cells are stained with an anti-rabies antibody conjugated to FITC. The antibody titer is defined as the reciprocal of the serum dilution, which reduces the challenge virus to 1FFD_50_. The titers obtained are approximately equal to those measured by the MNT. The rapidity with which the test can be carried out and the ease with which several samples may be tested at a time make it suitable for routine diagnosis. The demonstration of antibody in the serum in the absence of a history of vaccination for rabies or in CSF offers indirect evidence of rabies infection. The RFFIT has been shown to be slightly more sensitive than the MNT for detecting Virus Neutralizing antibodies (VNAs) in post-vaccinal sera.[36]

Fluorescence Antibody Virus Neutralization Test (FAVN)

This test, which is an adaptation of RFFIT, was developed by Cliquet *et al.* in 1998.[37] In this test, each patients diluted serum is placed in four wells of a microtiter plate containing LD_50_ live virus in cell line and each well is scored as having virus present or no virus after a 40-h incubation period. Results obtained with this test showed good agreement with the MNT and RFFIT. According to the authors, distinguishing negative sera from the positive with low titers of antibody is easier with the FAVN as compared to RFFIT. Hostnik has described a modification of this test in which the monoclonal anti-rabies antibodies and a peroxidase anti-mouse conjugate were used instead of a fluorescein-conjugated anti-rabies antibody.[38]

The Indirect immunofluorescence assay (IFA)

The IFA is based on the interaction between antibody and viral antigen present in smears of infected mouse brain or cell cultures. The bound antibody is visualized using a fluorescein-labeled species-specific antiserum directed against one or more immunoglobulins classes. The slide is examined under a fluorescent microscope. The reaction is graded by the amount and brilliance of fluorescence. The IFA is reported to be more sensitive than the neutralization test (NT) in detecting antibody early in the immune response, making it valuable for diagnosis.[39,40] The IFA can also be used in seroepidemiological surveys. It is a rapid test and is economical and easy to perform. However, this test does not directly correlate with NT when post-vaccinal sera are assayed. While Thomas *et al.* and Gispen and Sathof reported the IFA to be more sensitive than NT, several other investigators found NT to be better.[41–44] Madhusudhana *et al.*, from South India, reported a sensitivity of 97.2% and specificity of 97.9% for IFA.[45] A comparison of the two techniques showed that the relative sensitivity of the two test systems might be dependent on the vaccine used for immunization.[45] The IFA antibody titers of the sera from individuals vaccinated with duck embryo or nerve tissue vaccines were higher than those from individuals vaccinated with human diploid vaccine. While neutralizing antibody titers were measured, the converse was true. The large intracytoplasmic inclusions observed in rabies infected monolayers or brain impression smears by fluorescent antibody techniques, are due to antibody binding with the nucleocapsid proteins of the rabies virus. Antibody to viral glycoprotein stains infected cell membranes with little or no cytoplasmic staining

Membrane staining is not easily observed in acetone-fixed preparations and it would be difficult to detect by the customary IFA technique. Relative insensitivity in detecting antiglycoprotein antibody may lead to an inaccurate estimate of the protection afforded by immunization, making the IFA unsuitable for vaccine efficacy trials.[36]

The attempts for *ante mortem* diagnosis of rabies using clinical specimens may not be always successful, and negative tests do not rule out the possibility of rabies. In contrast to *ante mortem* diagnosis of rabies, *post mortem* diagnosis is more sensitive and specific. Negri bodies are present in more than 70% of infected human or animal brains [Figure 1B Inset]. More sensitive and specific tests include direct immunofluorescence test carried out on fresh brain smears.[46] The detection of rabies viral antigen in brain specimens can also be accomplished by ELISA based techniques such as rapid rabies enzyme imunodiagnosis (RREID) [Figure 2B] or recently developed dot-blot immunoassay.[47,48] The detection of immune complexes to rabies antigens may be used as one of the techniques for rapid ante-mortem diagnosis of human rabies. Recently, an ELISA test based on monoclonal antibodies to rabies nucleoprotein (N) and glycoprotein (G) was developed to detect immune complexes in rabies N and G proteins.[49] Studies have revealed the presence of rabies specific immune complexes in cerebrospinal fluid (CSF) of patient with paralytic rabies, which could help in *ante-mortem* diagnosis. Rabies virus antigen could be detected by agglutination on a glass slide using latex particles coated with gamma globulin. Recently Kasempimolporn *et al.* developed a simple and inexpensive test based on latex agglutination (LA) for rabies virus antigen detection in the saliva of live dogs.[50]

## Treatment and Management of Rabies Patients

Human rabies is still considered as cent percent fatal, although some cases of recovery have been reported form time to time.[51–53] The most recent case is a 15-year-old girl from USA who was treated by the induction of prolonged coma and by administering anesthetic, Ketamine and managed in intensive care unit.[1] This is the first case of recovery reported in an unvaccinated individual, whereas earlier cases had partial or full course of post-exposure vaccination. Despite such reports, rabies is still considered a fatal disease and patients who have already developed symptoms should be managed conservatively. Although some antiviral drugs such as ribavirine are found effective in experimental animals, they were ineffective in treating human cases.[54] Same results have been observed with Interferon[55] and high doses of intrathecally administered rabies immunoglobulin.[56] Cases of furious rabies should be isolated and sedated and allowed to depart his/her life peacefully. Cases of paralytic rabies pose a diagnostic and management problem in Intensive Care Units (ICUs). Clinical diagnosis is difficult and laboratory tests may or may not be conclusive. Giving the benefit of doubt, such patients should be treated in ICUs to give a chance for recovery of cases other than rabies. Medical and paramedical persons treating such suspected cases of rabies should take precautions not to get exposed to saliva and other secretions of the patient by direct contact. Although the transmission of rabies in such circumstances is very remote, it is advisable that treating physicians and nurses receive a recommended course of pre-exposure rabies vaccination as prophylaxis.

## Prevention of Rabies

Although rabies is virtually 100% fatal once the symptoms develop, it can be effectively managed if appropriate post-exposure treatment is instituted immediately after exposure. The post-exposure prophylaxis consists of three important ingredients viz. local wound treatment, passive immunization with rabies immunoglobulins and active immunization with rabies vaccines. All these are equally important and treatment failures have occurred due to neglecting any of the procedures, particularly passive immunization.

Local wound treatment should be instituted soon after the exposure. The bite wound(s) should be flushed with running tap water and washed with plenty of soap or detergent for at least 10 min. This removes the virus mechanically as well as inactivates the virus to a great extent. This should be followed with applying a local antiseptic such as Povidone Iodine. Suturing should be avoided as far as possible and if deemed necessary for surgical reasons, should be done only after infiltrating the wound with anti rabies immunoglobulin.

The further management of the bite depends on the degree or severity of exposures and risk of developing rabies. The present categorization of exposures and the required treatment procedures that is universally followed is as per the national guideline, the details of which can be accessed from the web site of Association for Prevention and Control of rabies in India (APCRI): www.apcri.org.

### Vaccination

The manufacture and use of outdated nerve-tissue-derived Semple vaccine has been discontinued in India since January 2005. Highly potent and safe cell culture vaccines are available for post-exposure treatment for almost 3 decades. These are the Human Diploid Cell Vaccine (HDCV), Purified Chick Embryo Cell vaccine (PCEC, Rabipur) and Purified Vero cell Rabies vaccine (PVRV, Verorab, Abhayrab). Recently, a highly purified duck embryo vaccine (PDEV, Vaxirab) was introduced in India. Five doses of vaccine must be administered on day 0, 3, 7, 14 and 28 intramuscularly over the deltoid region. The dosage schedule is same for all age groups and there is no contraindication for rabies vaccination. Sudarshan *et al.* assessed the safety of post-exposure rabies immunization and immunogenicity of purified vero cell rabies vaccine (PVRV) during pregnancy and found it to be safe and immunogenically efficacious. None of the women experienced any adverse side effects to the vaccine.[57] As cell culture vaccines are expensive and may not be economical for use in the developing countries, cost-effective intradermal regimens have been developed, which were approved by WHO in 1992.[58] Recently, the Government of India has also approved these regimens. These regimens reduce the cost of the treatment by almost 60%. In a recent WHO expert meeting on rabies, a modified Thai Red Cross Regimen consisting of administering 0.1 ml of PCEC or PVRV on both sides of the deltoid region, intradermally on day 0, 3 and 7 and at one side on day 28 was recommended.[59]

### Passive immunization

This is one aspect of post-exposure prophylaxis (PEP) that is often neglected by the medical practitioners. The presently available rabies vaccines induce protective levels of antibodies usually 10-14 days after initiating the course. Some cases of category III exposures may have very short incubation period and in such cases the mere administration of vaccine will not prevent rabies, as the virus would have reached CNS before the formation of sufficient quantities of neutralizing antibodies. Therefore, WHO advocates passive immunization with rabies immunoglobulin (RIG) in all category III exposures There are two types of RIGs presently available viz. highly purified and enzyme-refined equine rabies immunoglobulin (ERIG) and human rabies immunoglobulin (HRIG). The recommended dosage is 40 IU/kg body weight of ERIG and 20 IU/kg body weight of HRIG. The calculated dose should be infiltrated as much as possible locally into and around the wound and remaining if any should be administered IM in the gluteal region. The ERIGs should be administered only after a proper sensitivity test, although the incidence of anaphylactic reactions following the administration of present day ERIGs is very rare, (around 0.001%).[60] Other adverse reactions akin to serum sickness may be observed more frequently, but these tend to be mild and usually do not require any medication. During the recent Indian survey, it was startling to note that less than 2% of category III exposures received RIG. Most of the treatment failures encountered are due to lack of timely administration of RIG. At the neurological services of NIMHANS (a specialty center), Bangalore, some of the patients with rabies had received only a course of Cell Culture Vaccine (CCV) after the bite but not RIG for various reasons. Therefore, there is a need for an extensive campaign for the use of RIGs in our country. Moreover, rabies immunoglobulin should be strictly administered to all HIV/AIDS patients presenting with category II and III exposure in developing countries where CD4 counting is unreachable.[61]

### Pre-exposure vaccination

This is recommended to people who are at continued risk of exposure to rabies such as veterinarians, laboratory workers handling rabies virus, dog catchers and forest rangers, medical and paramedical people treating rabies patients in ICU. Three doses of any modern rabies vaccine can be administered on days 0, 7 and 28. A booster dose is recommended to people at continued risk.

### Vaccination after re-exposure

As per the WHO recommendation, 2 doses of any modern vaccine is required on days 0 and 3 for people who are re-exposed to rabies and have taken a course of complete post-exposure treatment with any of the modern cell culture vaccine any time in the past. There is no need for administration of rabies immunoglobulin. Incorporation of intradermal vaccine into childhood immunization schedules has been proposed for developing countries.[62] Hatz *et al.* recommended the pre-immunization to travelers visiting areas where canine rabies is enzootic and access to appropriate medical care is limited.[63]

The intramuscular route is recommended for rabies pre exposure vaccination if the antimalarial Chloroquine phosphate is being administered concurrently. This recommendation was made after a Peace Corps Volunteer died of rabies despite having completed a full intradermal (ID) pre-exposure series 6 months previously.[64]

In the era of wide international travel, neurologists, internists and medical practitioners in critical care management are confronted with cases of rabies masquerading as other neurological illness such as peripheral neuropathy. Similarly, in psychiatric practice, a case diagnosed as manic psychosis could in reality be one of the types of rabies. The knowledge of the technical modalities for antemortem diagnosis may assist in planning the hospital management and post-exposure prophylaxis of the treating medical team. Although the clinical outcome of rabies still remains grim, the pre-exposure and post-exposure vaccination in combination with passive immunization is likely to change the clinical outcome favorably.
